# Selective serotonin reuptake inhibitors (SSRIs) for stroke recovery

**DOI:** 10.1590/1516-3180.20131313T1

**Published:** 2013-06-01

**Authors:** Gillian E. Mead, Cheng-Fang Hsieh, Rebecca Lee, Mansur A. Kutlubaev, Anne Claxton, Graeme J. Hankey, Maree L. Hacklett

## Abstract

**BACKGROUND:**

Stroke is the major cause of adult disability. Selective serotonin reuptake inhibitors (SSRIs) have been used for many years to manage depression. Recently, small trials have demonstrated that SSRIs might improve recovery after stroke, even in people who are not depressed. Systematic reviews and meta-analyses are the least biased way to bring together data from several trials. Given the promising effect of SSRIs on stroke recovery seen in small trials, a systematic review and meta-analysis is needed.

**OBJECTIVE:**

To determine whether SSRIs improve recovery after stroke, and whether treatment with SSRIs was associated with adverse effects.

**METHODS:**

*Search methods:* We searched the Cochrane Stroke Group Trials Register (August 2011), Cochrane Depression Anxiety and Neurosis Group Trials Register (November 2011), Cochrane Central Register of Controlled Trials (CENTRAL) (The Cochrane Library 2011, Issue 8), MEDLINE (from 1948 to August 2011), EMBASE (from 1980 to August 2011), CINAHL (from 1982 to August 2011), AMED (Allied and Complementary Medicine) (from 1985 to August 2011), PsycINFO (from 1967 to August 2011) and PsycBITE (Pyschological Database for Brain Impairment Treatment Efficacy) (March 2012). To identify further published, unpublished and ongoing trials we searched trials registers, pharmaceutical websites, reference lists, contacted experts and performed citation tracking of included studies.

*Selection criteria:* We included randomized controlled trials that recruited stroke survivors (ischaemic or haemorrhagic) at any time within the first year. The intervention was any SSRI, given at any dose, for any period. We excluded drugs with mixed pharmacological effects. The comparator was usual care or placebo. In order to be included, trials had to collect data on at least one of our primary (dependence and disability) or secondary (impairments, depression, anxiety, quality of life, fatigue, healthcare cost, death, adverse events and leaving the trial early) outcomes.

*Data collection and analysis:* We extracted data on demographics, type of stroke, time since stroke, our primary and secondary outcomes, and sources of bias. For trials in English, two review authors independently extracted data. For Chinese papers, one review author extracted data. We used standardized mean differences (SMD) to estimate treatment effects for continuous variables, and risk ratios (RR) for dichotomous effects, with their 95% confidence intervals (CIs).

**MAIN RESULTS:**

We identified 56 completed trials of SSRI versus control, of which 52 trials (4059 participants) provided data for meta-analysis. There were statistically significant benefits of SSRI on both of the primary outcomes: RR for reducing dependency at the end of treatment was 0.81 (95% CI 0.68 to 0.97) based on one trial, and for disability score, the SMD was 0.91 (95% CI 0.60 to 1.22) (22 trials involving 1343 participants) with high heterogeneity between trials (I2 = 87%; P < 0.0001). For neurological deficit, depression and anxiety, there were statistically significant benefits of SSRIs. For neurological deficit score, the SMD was -1.00 (95% CI -1.26 to -0.75) (29 trials involving 2011 participants) with high heterogeneity between trials (I2 = 86%; P < 0.00001). For dichotomous depression scores, the RR was 0.43 (95% CI 0.24 to 0.77) (eight trials involving 771 participants) with high heterogeneity between trials (I2 = 77%; P < 0.0001). For continuous depression scores, the SMD was -1.91 (95% CI -2.34 to -1.48) (39 trials involving 2728 participants) with high heterogeneity between trials (I2 = 95%; P < 0.00001). For anxiety, the SMD was -0.77 (95% CI -1.52 to -0.02) (eight trials involving 413 participants) with high heterogeneity between trials (I2 = 92%; P < 0.00001). There was no statistically significant benefit of SSRI on cognition, death, motor deficits and leaving the trial early. For cognition, the SMD was 0.32 (95% CI -0.23 to 0.86), (seven trials involving 425 participants) with high heterogeneity between trials (I2 = 86%; P < 0.00001). The RR for death was 0.76 (95% CI 0.34 to 1.70) (46 trials involving 3344 participants) with no heterogeneity between trials (I2 = 0%; P = 0.85). For motor deficits, the SMD was -0.33 (95% CI -1.22 to 0.56) (two trials involving 145 participants). The RR for leaving the trial early was 1.02 (95% CI 0.86 to 1.21) in favour of control, with no heterogeneity between trials. There was a non-significant excess of seizures (RR 2.67; 95% CI 0.61 to 11.63) (seven trials involving 444 participants), a non-significant excess of gastrointestinal side effects (RR 1.90; 95% CI 0.94 to 3.85) (14 trials involving 902 participants) and a non-significant excess of bleeding (RR 1.63; 95% CI 0.20 to 13.05) (two trials involving 249 participants) in those allocated SSRIs. Data were not available on quality of life, fatigue or healthcare costs. There was no clear evidence from subgroup analyses that one SSRI was consistently superior to another, or that time since stroke or depression at baseline had a major influence on effect sizes. Sensitivity analyses suggested that effect sizes were smaller when we excluded trials at high or unclear risk of bias. Only eight trials provided data on outcomes after treatment had been completed; the effect sizes were generally in favour of SSRIs but CIs were wide.

**AUTHORS’ CONCLUSIONS:**

SSRIs appeared to improve dependence, disability, neurological impairment, anxiety and depression after stroke, but there was heterogeneity between trials and methodological limitations in a substantial proportion of the trials. Large, well-designed trials are now needed to determine whether SSRIs should be given routinely to patients with stroke.

**This is the abstract of a Cochrane Review published in the Cochrane Database of Systematic Reviews (CDSR) 2012, issue 11, Art. No. CD009286. DOI:** 10.1002/14651858.CD009286.pub2 (http://onlinelibrary.wiley.com/doi/10.1002/14651858.CD009286.pub2/abstract). For full citation and authors details, see reference 1

**The full text is freely available from:** http://www.cochranejournalclub.com/SSRIs-for-stroke-recovery-clinical/pdf/CD009286.pdf (this link may be temporary), or http://www.cochranejournalclub.com/SSRIs-for-stroke-recovery-clinical/pdf/CD009286_abstract.pdf
